# Coping under pressure: exploring the role of resilience and emotional regulation in enhancing athletes’ wellbeing amid intense training

**DOI:** 10.3389/fpsyg.2026.1828846

**Published:** 2026-07-20

**Authors:** Endong Qiao, Anping Chen

**Affiliations:** School of Physical Education, Shanxi University, Shanxi, China

**Keywords:** coping strategies, emotional regulation, mental resilience, training intensity, wellbeing

## Abstract

**Introduction:**

This study examines the mediating and moderating mechanisms through which coping strategies influence athletes’ wellbeing in resilience training programs. Drawing on the Transactional Model of Stress and Coping, we propose that mental resilience and emotional regulation mediate the relationship between coping strategies and wellbeing, while training intensity strengthens these relationships.

**Methods:**

A moderated mediation model was developed and tested using data collected from 300 athletes participating in structured resilience training programs. The study examined the associations of problem-focused and emotion-focused coping strategies with wellbeing through the mediating roles of mental resilience and emotional regulation, and assessed whether training intensity moderated these pathways.

**Results:**

The findings supported the hypothesized model. Both mental resilience and emotional regulation significantly mediated the relationship between coping strategies and athletes’ wellbeing. In addition, training intensity significantly moderated the relationships between coping strategies and both mediators, with stronger associations observed among athletes engaged in higher-intensity training. The positive effects of coping strategies on wellbeing were therefore enhanced under conditions of greater training intensity.

**Discussion:**

The results highlight the importance of fostering effective coping strategies, mental resilience, and emotional regulation to improve athletes’ wellbeing. They further suggest that higher-intensity resilience training may amplify these beneficial effects. These findings provide practical guidance for optimizing resilience training programs and offer directions for future research on interventions designed to enhance athletes’ psychological wellbeing.

## 1 Introduction

Modern athletic environments are becoming increasingly aware of the need to promote athletes’ wellbeing as a means to sustain long-term performance and competitive advantage (Qi et al., 2024). Simultaneously, the physical and psychological demands associated with high-intensity training require athletes to employ effective coping strategies to maintain both performance and psychological wellbeing (Simpson et al., 2024; Gray et al., 2024). Therefore, developing coping strategies that enhance mental resilience and emotional regulation is essential to safeguard athletes’ wellbeing.

Although athletes face considerable challenges in managing both physical and psychological demands, organizations and sports programs need to equip them with effective coping strategies to handle these stressors. Coping strategies, specifically problem-focused and emotion-focused coping, are vital tools for athletes to navigate high-pressure environments (Algorani and Gupta, 2023). Problem-focused coping involves directly addressing the stressors, thereby reducing or eliminating their impact, while emotion-focused coping allows athletes to regulate their emotional responses, reducing emotional strain (Schoenmakers et al., 2015). Moreover, problem-focused coping may also facilitate emotional regulation by reducing uncertainty and enhancing athletes’ perceived control over stressful situations. As athletes actively address the source of stress, they are better able to minimize emotional distress and maintain psychological balance, thereby supporting more effective emotional regulation (Carroll, 2020; Gross, 1998; Beatty and Janelle, 2020). Both strategies are crucial in maintaining an athlete’s mental resilience—their ability to recover from setbacks—and emotional regulation, which stabilizes emotional responses in high-stakes situations. Without these psychological resources, athletes are more vulnerable to psychological fatigue, poorer wellbeing, and diminished performance (Algorani and Gupta, 2023; Cosma et al., 2020).

Despite the established benefits of coping strategies, the role of training intensity in influencing their effectiveness has been under-researched. Training intensity is a key variable that can either enhance or diminish the success of coping strategies in fostering wellbeing (Suchomel et al., 2021). Higher intensity levels introduce greater stress, pushing athletes to their physical and mental limits. At these times, coping mechanisms—if effectively employed—may become even more critical in preserving wellbeing by bolstering mental resilience and enhancing emotional regulation (Mendez, 2024). Although these relationships have received increasing attention, most existing evidence has been generated in Western sporting environments (Song et al., 2024; Tamir et al., 2024). Comparatively less research has examined whether the interaction between coping strategies and training intensity operates similarly among Chinese athletes, whose training environments are often characterized by intensive coaching practices, structured training systems, and unique sociocultural expectations. Consequently, the applicability of existing evidence to the Chinese athletic context remains insufficiently understood, warranting further investigation. However, under lower intensity, the same strategies may not be as necessary or impactful, creating a moderating effect that needs further exploration.

Building on the Transactional Model of Stress and Coping (Lazarus and Folkman, 1984), this study examines how coping strategies are associated with wellbeing, mediated by mental resilience and emotional regulation, with training intensity acting as a moderator. Mental resilience helps athletes bounce back from stressors, and emotional regulation ensures athletes can maintain composure under pressure. By examining the interplay among these variables, this research aims to provide deeper insights into the psychological processes that support wellbeing in competitive and high-intensity environments. More specifically, by examining these relationships among Chinese athletes, this study extends the applicability of the Transactional Model of Stress and Coping beyond its predominant Western contexts and contributes evidence regarding how coping strategies function under intensive training conditions within China’s sporting environment. Moreover, by considering training intensity as a moderator, the study explores the conditions under which coping strategies are most effective, shedding light on how athletes’ mental resources can be optimized in resilience training programs.

The study contributes to existing research in several ways. First, the study builds on the literature combining the Transactional Model of Stress and Coping (Lazarus and Folkman, 1984) with athlete wellbeing in the context of resilience training. While much has been written about stress management in athletes (Gray et al., 2024; Simpson et al., 2024), limited research has examined the combined effects of coping strategies, mental resilience, and emotional regulation in the same model. Furthermore, limited evidence has examined these relationships within the Chinese athletic context (Zuo and Bai, 2025), despite the distinctive training structures and competitive demands experienced by Chinese athletes. By addressing this contextual gap, the present study extends existing knowledge regarding the applicability of the Transactional Model of Stress and Coping across different sporting environments. By analyzing the role of coping strategies in conjunction with both psychological resilience and emotional regulation mechanisms, the study addresses the gaps in understanding how athletes maintain wellbeing under high-pressure conditions (Lazarus and Folkman, 1984).

Second, the study highlights the moderating influence of training intensity on the coping strategies–wellbeing relationship. The interaction between training intensity and coping mechanisms has been underexplored in sports psychology (Poulus et al., 2022). The current study further examines whether this relationship varies under different levels of training intensity, providing new insights into the conditions under which coping strategies are more strongly associated with wellbeing.

Third, the study broadens the scope of mediators by including mental resilience and emotional regulation as key psychological processes that mediate the relationship between coping strategies and wellbeing. By integrating both mediators within a single framework, the study also provides a stronger theoretical explanation of how coping strategies are translated into psychological wellbeing. Specifically, problem-focused coping not only assists athletes in addressing external stressors but also facilitates emotional regulation by increasing perceived control and reducing emotional arousal, thereby complementing the adaptive role of mental resilience (Beatty and Janelle, 2020; Gross, 1998). This dual mediation framework therefore offers a more comprehensive explanation of the psychological processes underlying athletes’ wellbeing in demanding training environments.

Lastly, given the mixed results in previous studies regarding the impact of coping strategies on wellbeing (Rose et al., 2023), the study explores both direct and indirect effects to fill this research gap. By incorporating training intensity as a moderator, the study offers a more nuanced understanding of the conditions under which coping strategies are most effective, contributing to the growing literature on stress, coping, and athlete wellbeing.

## 2 Literature review

### 2.1 Transactional model of stress and coping

The Transactional Model of Stress and Coping (Lazarus and Folkman, 1984) provides the foundation for understanding how individuals respond to stress. According to this model, stress is conceptualized as a transaction between an individual and their environment, where the individual appraises a situation as stressful or non-stressful, followed by an evaluation of available coping mechanisms. The model emphasizes two types of coping strategies: problem-focused coping, which involves addressing the source of stress directly, and emotion-focused coping, which focuses on managing the emotional responses to stressors (Gray et al., 2024; Simpson et al., 2024). Both types of coping play critical roles in how individuals manage high-pressure environments.

In the context of athletic performance, athletes constantly face stressors ranging from physical exhaustion to psychological strain due to competition, high training intensity, and external expectations (Poulus et al., 2022). Applying the transactional model to athletes, coping strategies become essential tools for managing the multifaceted stressors inherent in resilience training programs. Problem-focused coping might manifest in behaviors such as adapting training routines or addressing physical challenges, while emotion-focused coping could involve managing anxiety or frustration during competitions. These strategies are crucial for maintaining athletes’ mental health and optimizing their performance (Lazarus and Folkman, 1984).

### 2.2 Theoretical underpinning in the model

The theoretical underpinning of the model relies on the Transactional Model of Stress and Coping to explain the pathway from coping strategies to wellbeing. Coping strategies, when employed effectively, lead to higher levels of mental resilience and emotional regulation, both of which are critical in maintaining wellbeing. Mental resilience is particularly relevant as it refers to an athlete’s ability to “bounce back” from stress, which is a crucial skill during high-stakes training (Mendez, 2024). Emotional regulation further supports athletes by managing the emotional toll of competition and rigorous training schedules (Sun et al., 2023).

Furthermore, the inclusion of training intensity as a moderator enhances the model by demonstrating the variability in coping strategies’ effectiveness under different levels of stress. High training intensity represents a condition in which stress is amplified (Doron et al., 2024), requiring more robust coping mechanisms. The transactional model suggests that under more intense stressors, athletes must rely more heavily on coping mechanisms, which strengthens the relationships between coping strategies and the mediators (mental resilience and emotional regulation).

In summary, the transactional model allows us to explain how athletes process and respond to stressors in high-intensity environments. Coping strategies influence wellbeing indirectly through mental resilience and emotional regulation, and the level of training intensity moderates these pathways. This framework provides a comprehensive explanation of how stress, coping, and performance are interrelated in the context of resilience training, offering valuable insights into both psychological and physical wellbeing in athletes.

### 2.3 Relationship between coping strategies and wellbeing

Coping strategies refer to the cognitive and behavioral efforts individuals employ to manage stress and difficult situations (Lazarus and Folkman, 1984). These strategies are typically divided into two primary categories: problem-focused coping and emotion-focused coping. Problem-focused coping involves taking direct action to address the root cause of the stressor, such as developing a solution, changing one’s behavior, or seeking help (Schoenmakers et al., 2015). On the other hand, emotion-focused coping is aimed at managing the emotional distress that accompanies stressful situations. It includes strategies like reappraising the situation, seeking emotional support, or using relaxation techniques to reduce stress levels (Gray et al., 2024; Simpson et al., 2024).

The distinction between these two strategies is critical in understanding how individuals respond to different types of stressors. Problem-focused coping is typically more effective when the individual has some control over the situation and can make changes to alter the outcome (Carroll, 2020). For instance, athletes might adjust their training regimen or seek additional coaching to address performance-related stress. Conversely, emotion-focused coping is more effective in situations where the stressor is beyond the individual’s control, such as injury or external pressures from competition (Schoenmakers et al., 2015). In these cases, athletes may rely on managing their emotional responses, such as anxiety or frustration, rather than changing the situation itself.

The relationship between coping strategies and wellbeing is well-established in psychological literature (Galiana et al., 2020). Wellbeing, often conceptualized as a state of mental health and emotional stability (Murphy et al., 2020), can be significantly influenced by the coping mechanisms individuals use to manage stress. When effective coping strategies are employed, individuals are better able to maintain a positive mental state, even in the face of adversity (Potts et al., 2023). Problem-focused coping, for example, has been associated with long-term improvements in wellbeing as individuals gain a sense of control over their circumstances and successfully address stressors (Carroll, 2020). Emotion-focused coping, while beneficial in the short term for reducing emotional distress, can sometimes lead to maladaptive outcomes if overused, particularly when it results in avoidance or denial of the stressor (Poulus et al., 2022).

In the context of sports, recent literature supports the positive impact of coping strategies on athletes’ wellbeing. For instance, a study by Cosma et al. (2020) found that athletes who used problem-focused coping to manage the demands of training and competition reported higher levels of psychological wellbeing and lower levels of burnout compared to those who predominantly relied on emotion-focused strategies. Similarly, Mendez (2024) demonstrated that athletes with higher levels of mental resilience—often developed through effective coping strategies—were better able to maintain emotional stability and positive wellbeing during high-pressure situations, such as competitions or intense training periods.

In the related stream, Leguizamo et al. (2021) explored the relationship between coping strategies and wellbeing in elite athletes and found that those who effectively managed both the problem and emotional aspects of stress were more likely to report higher satisfaction with life and fewer symptoms of anxiety and depression. Their research highlights the importance of a balanced approach to coping, where athletes use both problem-focused and emotion-focused strategies depending on the situation’s demands. In summary, coping strategies play a crucial role in influencing wellbeing, particularly in high-stress environments like sports. Problem-focused coping generally leads to better outcomes when athletes can control the stressors, while emotion-focused coping is essential for managing emotional responses to unavoidable stressors. Hence, the first hypothesis states:

*H1*: There is a significant positive relationship between coping strategies—(a) problem-focused; (b) emotion-focused—and wellbeing.

### 2.4 Relationship between mental resilience and wellbeing

Mental resilience refers to an individual’s ability to adapt to stress, adversity, or challenging situations while maintaining or quickly regaining a positive mental state (Petersen, 2024). It encompasses both cognitive and emotional aspects (Ryff and Singer, 2003), enabling individuals to “bounce back” from setbacks and continue functioning effectively under pressure. Mental resilience involves a dynamic process of coping and adaptation, where individuals draw upon internal and external resources to manage stressors (Fletcher and Sarkar, 2013). According to Petersen (2024), mental resilience is not a fixed trait but can be developed and enhanced through training, experience, and coping mechanisms.

In high-pressure environments like sports, mental resilience becomes an essential skill. Athletes frequently encounter stressful situations such as intense training sessions, competition pressures, injuries, and external expectations. Those with higher levels of mental resilience are better equipped to cope with these stressors, reducing the negative psychological impact and preserving their mental health (Vella et al., 2021). Besides, mental resilience acts as a buffer against stress, allowing individuals to maintain focus, motivation, and emotional stability even when faced with adverse conditions (Dumsday and Yeoh, 2023). Further, individuals with higher levels of mental resilience tend to experience greater overall wellbeing because they are able to prevent stress from overwhelming them. Instead of succumbing to negative emotions like anxiety or frustration, they use their resilience to maintain a positive outlook, even during challenging times (Mendez, 2024).

Recent research further supports this connection in the context of sports. For example, Gupta and McCarthy (2022) found that athletes with higher mental resilience were more likely to report positive psychological wellbeing, as they were able to handle the stress of competition without experiencing burnout or emotional exhaustion. Similarly, Husain et al. (2024) demonstrated that elite athletes with high resilience had lower levels of stress and anxiety, contributing to a more positive emotional state. These studies highlight the protective role of mental resilience in safeguarding an individual’s wellbeing by preventing stress from having a prolonged negative impact. Moreover, mental resilience enables athletes to approach stress as an opportunity for growth rather than a threat to their wellbeing (Gupta and McCarthy, 2022). This adaptive mindset helps athletes reframe challenges in a way that fosters personal development and psychological strength (Dumsday and Yeoh, 2023). When individuals perceive stressful situations as manageable, they are less likely to experience detrimental effects on their wellbeing. Consequently, mental resilience not only helps athletes cope with stress but also contributes to sustained emotional wellbeing over time. Hence, the second hypothesis states:

*H2*: There is a significant positive relationship between mental resilience and wellbeing.

### 2.5 Relationship between emotional regulation and wellbeing

Emotional regulation refers to the process by which individuals monitor, evaluate, and modify their emotional responses to meet situational demands and personal goals (Beatty and Janelle, 2020). Emotional regulation involves both the suppression of negative emotions and the enhancement of positive ones, allowing individuals to maintain control over their emotional states in the face of stress or adversity. Further, it can be conscious, such as choosing to reframe a situation to reduce anxiety, or unconscious, where the individual automatically shifts focus away from distressing stimuli (Gray et al., 2024; Simpson et al., 2024).

In the context of athletic performance, emotional regulation is crucial for maintaining focus, composure, and motivation, particularly during high-pressure situations like competitions or intense training sessions. Athletes who can effectively regulate their emotions are better able to prevent feelings of frustration, anxiety, or anger from interfering with their performance. Emotional regulation also helps in sustaining mental clarity and decision-making under stress, which are essential for successful outcomes in sports (Costa et al., 2020). There is a wide agreement that individuals who employ effective emotional regulation strategies tend to experience more positive emotional states, as they can prevent negative emotions from overwhelming them (Doorley et al., 2022). In contrast, poor emotional regulation can lead to emotional exhaustion, anxiety, and decreased overall wellbeing (Doorley et al., 2022).

A review of recent literature highlights the significance of emotional regulation in the wellbeing of athletes. Teques et al. (2024) found that athletes with better emotional regulation skills reported higher levels of psychological wellbeing, as they were able to maintain emotional balance despite the pressures of competition. This suggests that emotional regulation acts as a protective mechanism, shielding athletes from the emotional toll of high-stress environments. Additionally, (Costa et al., 2020) demonstrated that emotional regulation not only enhances wellbeing but also contributes to improved athletic performance by allowing athletes to stay focused and motivated during challenging situations.

Moreover, the ability to regulate emotions has been shown to foster resilience, further promoting wellbeing. Beatty and Janelle (2020) found that individuals who are better at regulating their emotions are more likely to exhibit resilient behaviors, such as quickly bouncing back from setbacks and maintaining a positive outlook. This resilience, in turn, contributes to long-term psychological health and wellbeing. For athletes, emotional regulation enables them to handle the emotional highs and lows of their sport, from the thrill of victory to the disappointment of defeat, without experiencing lasting negative effects on their mental health (Hahn, 2021). Hence, the third hypothesis states:

*H3*: There is a significant positive relationship between emotional regulation and wellbeing.

### 2.6 Mediators: mental resilience and emotional regulation

The mediating roles of mental resilience and emotional regulation offer key insights into the pathways through which coping strategies influence wellbeing. In the Transactional Model of Stress and Coping (Lazarus and Folkman, 1984), coping strategies are essential in managing stress and preserving mental health, but their effectiveness often depends on intermediary processes like mental resilience and emotional regulation. These mediators explain how coping strategies translate into improved wellbeing by enhancing an individual’s capacity to handle stress and regulate emotional responses.

Mental resilience serves as a crucial mediator in the relationship between coping strategies and wellbeing. Individuals who adopt problem-focused coping strategies—actively addressing the source of stress—are more likely to build resilience over time (Carroll, 2020). Resilience allows athletes to not only recover from setbacks but also to thrive under pressure (Dumsday and Yeoh, 2023), maintaining their wellbeing despite the demands of high-intensity training. For example, by confronting the challenges of their sport head-on, athletes strengthen their ability to cope with future stressors, thereby experiencing higher levels of wellbeing. Emotional-focused coping, which involves managing the emotional impact of stressors (Schoenmakers et al., 2015), may also enhance resilience by reducing the immediate emotional burden, allowing individuals to approach stress more constructively.

Emotional regulation, similarly, plays a mediating role by managing the emotional responses that arise from stress, thereby safeguarding wellbeing. Coping strategies like emotion-focused coping are directly aimed at regulating emotions, helping individuals to maintain emotional balance in stressful environments (Algorani and Gupta, 2023). For athletes, effective emotional regulation allows them to stay composed under pressure, preventing emotional exhaustion and promoting mental clarity (Costa et al., 2020), both of which are crucial for long-term wellbeing. Whether through reframing negative emotions or finding ways to remain calm in stressful situations (Potts et al., 2023), emotional regulation acts as a bridge between the use of coping strategies and the preservation of wellbeing (Rose et al., 2023). Problem-focused coping can also enhance emotional regulation by reducing the stressor’s impact, which indirectly stabilizes emotions and contributes to wellbeing. Hence, the mediator hypotheses states:

*H4*: Mental resilience mediates the relationship between coping strategies—(a) problem-focused; (b) emotion-focused—and wellbeing.

*H5*: Emotional regulation mediates the relationship between coping strategies—(a) problem-focused; (b) emotion-focused—and wellbeing.

### 2.7 Moderator: training intensity

Training intensity refers to the physical and psychological demands placed on athletes during their training sessions, often characterized by the level of effort, duration, and frequency of workouts (Suchomel et al., 2021). In the context of the relationship between coping strategies and wellbeing, training intensity acts as a critical moderator that can either amplify or attenuate the effects of coping mechanisms. Under high-intensity training conditions, the demands placed on athletes are significantly elevated, necessitating more robust and effective coping strategies to manage the resulting stress and emotional strain (Doron et al., 2024). Conversely, in lower-intensity settings, the need for coping strategies may be less pronounced, as the stressors are more manageable (Peters et al., 2017).

The moderating role of training intensity can be understood through the lens of the Transactional Model of Stress and Coping (Lazarus and Folkman, 1984). According to this model, the effectiveness of coping strategies is contingent upon the nature and magnitude of the stressor. In high-intensity training environments, stress levels are heightened, placing greater demands on the individual’s psychological resources (Dumsday and Yeoh, 2023). Athletes under these conditions are more likely to benefit from problem-focused coping, which helps them address the challenges directly, and emotion-focused coping, which manages their emotional responses to the elevated stress. As the intensity increases, the relationship between coping strategies and wellbeing becomes stronger, as athletes rely more heavily on these strategies to maintain their mental and emotional health (Franchini, 2020).

**FIGURE 1:**
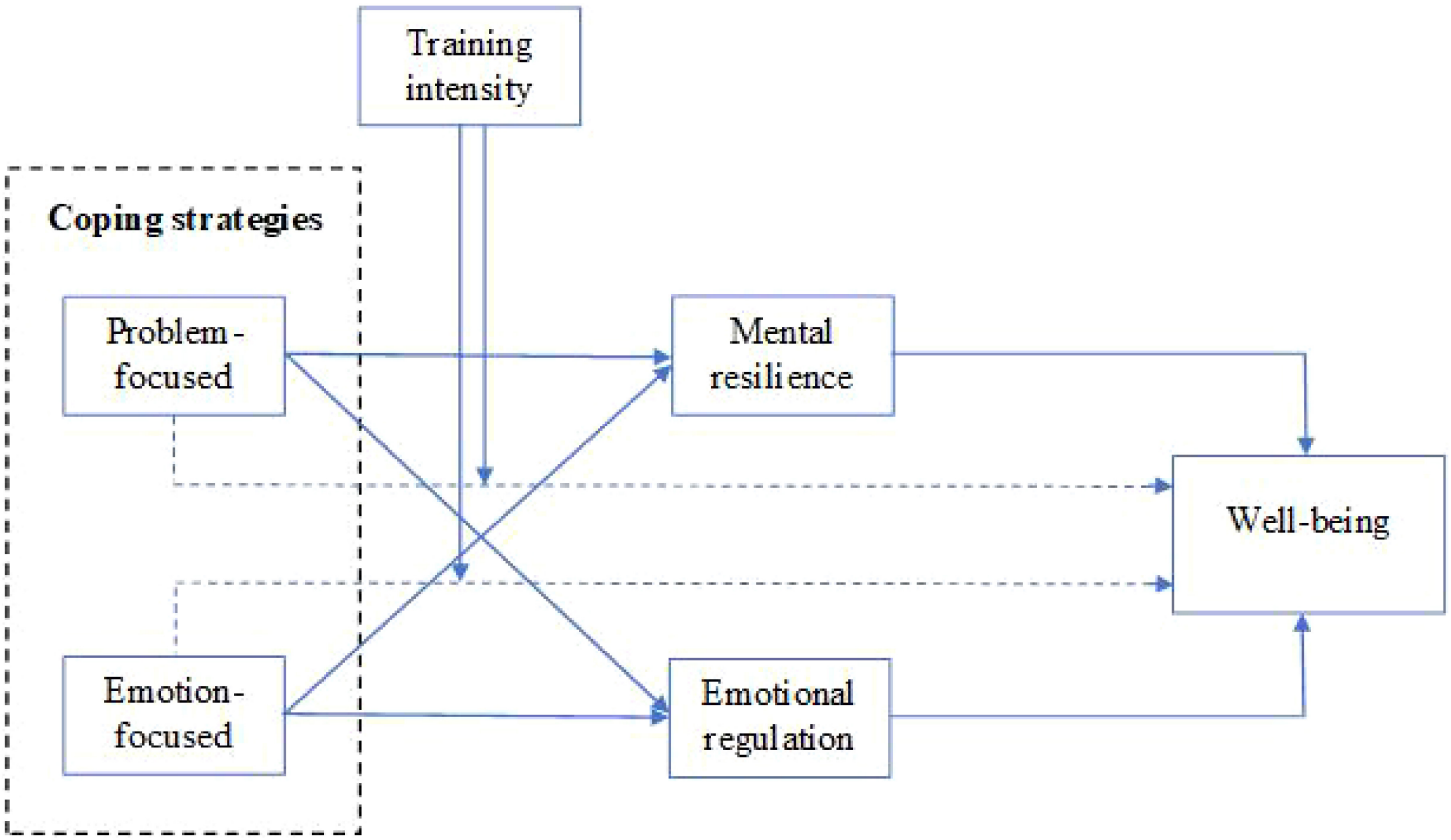
Conceptual model.

In this regard, training intensity can also influence the mediating roles of mental resilience and emotional regulation. Under high-intensity conditions, athletes who effectively use coping strategies are more likely to develop higher levels of mental resilience (Jones, 2024), as they repeatedly confront and adapt to challenging situations (Poulus et al., 2022). Similarly, the heightened emotional demands of intense training require stronger emotional regulation skills to prevent burnout and emotional fatigue (Costa et al., 2020). In such contexts, athletes who can successfully regulate their emotions and build resilience will experience greater wellbeing, making the moderating effect of training intensity a key variable in understanding how coping strategies operate under different conditions (Hut et al., 2021). In summary, training intensity moderates the relationship between coping strategies and wellbeing by influencing how much athletes rely on these strategies to manage stress. In higher-intensity training environments, the need for effective coping mechanisms, mental resilience, and emotional regulation is greater, thereby strengthening the relationship between these variables and overall wellbeing. Hence,

*H6*: Training intensity moderates the indirect effects of coping strategies—(a) problem-focused; (b) emotion-focused—on wellbeing through (i) mental resilience and (ii) emotional regulation, such that the mediating effects will be stronger under conditions of higher training intensity (see Figure 1).

## 3 Research methodology

The study adopted a quantitative, cross-sectional research design to examine the relationships among coping strategies, mental resilience, emotional regulation, training intensity, and athletes’ wellbeing. The study involved athletes participating in structured resilience training programs conducted at sports training centers across Shanxi Province, China. Participants were selected based on specific criteria, including their involvement in high-intensity training environments and their exposure to competitive sports, ensuring the relevance of the sample to the study’s objectives. To gather data, a convenience sampling technique was employed, targeting athletes from various training centers who met the criteria. This technique was deemed appropriate for the study due to its practicality in accessing athletes across multiple locations and ensuring a diverse range of participants in terms of experience and training intensity. Given the focus on psychological resilience and coping strategies in high-pressure environments, convenience sampling allowed for efficient data collection while capturing relevant data from athletes in demanding training scenarios.

The structured resilience training programs were implemented as standardized psychological skills development programs integrated into the athletes’ regular training schedules. The programs consisted of weekly 60–90-min sessions conducted over an 8-week period and focused on developing adaptive coping strategies, psychological resilience, emotional regulation, stress management, and performance enhancement techniques. To minimize potential intervention-related bias, all participating athletes completed programs with a comparable structure, duration, and training objectives under the supervision of qualified coaching staff and sport psychology practitioners.

A total of 350 questionnaires were administered across several training programs, but after screening for incomplete responses and ensuring that participants met the selection criteria, 300 valid responses were used for data analysis.

### 3.1 Participants

The final sample included athletes from different sports disciplines and age groups. Participants were recruited from sports training centers located in Taiyuan, Datong, Changzhi, and Jinzhong, Shanxi Province, China. The sample comprised athletes participating in both individual sports (55%), including athletics, swimming, badminton, table tennis, and martial arts, and team sports (45%), including basketball, football, and volleyball. Regarding competitive level, 45% were university-level athletes, 35% competed at the provincial level, and 20% competed at the national level, thereby providing a diverse representation of competitive experience.

In terms of demographics, 65% of the participants were male and 35% were female, ensuring representation of women and men. The participants ranged in age from 18 to 35 years (*M* = 26.8 years, SD = 4.2). The age range of participants was between 18 and 35 years, with 39% falling between 18 and 25 years, 45% between 26 and 30 years, and 16% between 31 and 35 years. Participants had varying levels of experience in their respective sports, with 57% involved in competitive sports for over 5 years, and 43% having been involved for less than 5 years. This range of demographics shown in Table 1 provided a broad perspective on the coping strategies, mental resilience, and emotional regulation abilities of athletes in different stages of their athletic careers, thereby enhancing the representativeness of the sample across different sporting disciplines and competitive levels while acknowledging the inherent limitations associated with convenience sampling.

**TABLE 1:** Participants characteristics.

Characteristic	Category	*n*	%
Gender	Male	195	65.0
	Female	105	35.0
Age	18–25	117	39.0
	26–30	135	45.0
	31–35	48	16.0
Sport type	Individual	165	55.0
	Team	135	45.0
Competitive level	University	135	45.0
	Provincial	105	35.0
	National	60	20.0
Experience	< 5 years	129	43.0
	≥ 5 years	171	57.0

## 4 Measures

The study utilized a 5-point Likert scale ranging from 1 (“strongly disagree”) to 5 (“strongly agree”) to assess the key variables. The questionnaire was originally developed in English and translated into Chinese using the Brislin (1980) back-translation procedure. Two bilingual experts independently translated and back-translated the instrument to ensure conceptual equivalence, and the translated version was subsequently reviewed by two sport psychology scholars for linguistic clarity and contextual appropriateness. Minor wording refinements were made before the final administration. The adequacy of the translated instrument was subsequently supported through the measurement model assessment, including construct reliability, convergent validity, and discriminant validity.

Coping strategies were measured using eight adapted items from Lazarus and Folkman (1984), with both problem-focused coping (e.g., “When faced with challenges, I actively work on solving the problem.”) and emotion-focused coping (e.g., “I try to change the way I feel about stressful situations.”) included. Mental resilience was assessed using five adapted items from Fletcher and Sarkar (2013), capturing participants’ ability to bounce back from adversity (e.g., “I am able to recover quickly after facing adversity.”). Emotional regulation was measured using five adapted items from Gross (1998), evaluating how participants manage their emotional responses (e.g., “I am able to stay calm when I feel stressed.”). Wellbeing was measured with five adapted items from Ryff and Singer (2003), focusing on overall emotional stability (e.g., “I feel mentally balanced and in control of my emotions.”). Finally, training intensity was measured using four adapted items from Peters et al. (2017), assessing the physical and mental demands of participants’ training (e.g., “The intensity of my training sessions challenges me both physically and mentally.”). The final questionnaire comprised 27 measurement items, excluding demographic questions. Each scale was adapted to fit the specific context of athletes participating in structured resilience training programs.

### 4.1 Procedure and ethical considerations

Data were collected between September and November 2024 through paper-based questionnaires administered at participating sports training centers immediately following scheduled training sessions. Before questionnaire administration, the researchers explained the purpose of the study, emphasized the voluntary nature of participation, assured respondents that their responses would remain anonymous and confidential, and informed them that they could withdraw from the study at any stage without penalty. Informed consent was obtained from all participants before data collection. The study was conducted in accordance with the ethical principles of the Declaration of Helsinki and received approval from the institutional ethics committee of the corresponding author’s university.

### 4.2 Data analysis plan

Data were analyzed using SmartPLS 4.0. Prior to structural model evaluation, the dataset was screened for missing values, incomplete responses, and data entry errors. Descriptive statistics and correlation analyses were conducted to summarize the sample characteristics and examine the relationships among the study variables. The distribution of the data was also assessed before model estimation. Partial Least Squares Structural Equation Modeling (PLS-SEM) was employed because the study was prediction-oriented and involved a complex research model incorporating multiple mediating and moderating relationships, making it an appropriate analytical technique (Hair et al., 2019). Furthermore, PLS-SEM is well suited for studies with relatively small to medium sample sizes and does not require strict assumptions of multivariate normality (Henseler et al., 2015), making it appropriate for the present dataset (*n* = 300). The measurement model was first evaluated by examining indicator reliability, internal consistency reliability (Cronbach’s alpha and composite reliability), convergent validity (average variance extracted), discriminant validity (heterotrait-monotrait ratio), and multicollinearity (variance inflation factor). Subsequently, the structural model was assessed using a bootstrapping procedure with 5,000 resamples to estimate path coefficients, indirect effects, moderation effects, confidence intervals, and significance levels for the hypothesized relationships.

## 5 Measurement model

### 5.1 Descriptive statistics

First of all, descriptive statistics of the study variables are assessed. Table 2 presents the descriptive statistics for the study variables based on the responses of the 300 athletes included in this study. Overall, the participants reported moderate to relatively high levels of problem-focused coping, emotion-focused coping, mental resilience, emotional regulation, training intensity, and wellbeing. The standard deviations indicate acceptable variability across responses, while the observed minimum and maximum values demonstrate that the full range of the measurement scale was represented, supporting the suitability of the data for subsequent PLS-SEM analysis.

**TABLE 2:** Descriptive statistics.

Variable	Items	Min	Max	Mean	SD
Problem-focused coping	4	1.00	5.00	3.89	0.71
Emotion-focused coping	4	1.00	5.00	3.72	0.75
Mental resilience	5	1.00	5.00	3.95	0.68
Emotional regulation	5	1.00	5.00	3.83	0.73
Training intensity	4	1.00	5.00	3.91	0.70
Wellbeing	5	1.00	5.00	3.88	0.69

### 5.2 Collinearity statistics (variance inflation factor: VIF)

Table 3 presents the variance inflation factor (VIF) values for each construct, assessing potential multicollinearity issues within the model. Generally, VIF values above *5* indicate problematic levels of collinearity (Hair et al., 2019), while values below *5* suggest that multicollinearity is not a concern. In this table, the VIF values for all constructs (emotion-focused, problem-focused, emotional regulation, mental resilience, training intensity, and wellbeing) fall below the critical threshold of *5*, with the highest VIF value being *2.115* for emotional regulation. This indicates that the constructs are not highly correlated with each other, and multicollinearity does not pose a significant issue in the model. Therefore, the structural equation model is reliable in terms of collinearity and supports the validity of further analysis using PLS-SEM.

**TABLE 3:** Collinearity statistics (variance inflation factor: VIF).

Construct	EF	PF	ER	MR	TI	WB
Emotion-focused			1.111	1.097		1.222
Problem-focused			1.234	1.472		1.189
Emotional regulation						2.115
Mental resilience						1.394
Training intensity			2.000	1.982		1.119
Wellbeing						

EF, emotion-focused; PF, problem-focused; ER, emotional regulation; MR, mental resilience; TI, training intensity; WB, wellbeing.

### 5.3 Construct reliability and validity

Table 4 evaluates the reliability and validity of the constructs based on Cronbach’s alpha, composite reliability (rho_c), and the average variance extracted (AVE). According to Hair et al. (2019), Cronbach’s alpha and composite reliability values above 0.7 indicate good reliability, while an AVE value greater than 0.5 reflects adequate convergent validity. In this table, all constructs (emotion-focused, problem-focused, emotional regulation, mental resilience, training intensity, and wellbeing) exceed these thresholds. For instance, emotion-focused coping shows a Cronbach’s alpha of 0.888 and an AVE of 0.721, demonstrating both strong internal consistency and convergent validity. The same applies to the other constructs, where composite reliability values range from 0.883 to 0.937, and AVE values are all above 0.666. These results confirm the construct reliability and convergent validity of the measures used in this study, ensuring that the items reliably reflect the intended constructs.

**TABLE 4:** Construct reliability and validity.

Construct	Cronbach’s alpha	Composite reliability (rho_a)	Composite reliability (rho_c)	Average variance extracted (AVE)
Emotion-focused	0.888	0.900	0.910	0.721
Problem-focused	0.917	0.920	0.937	0.689
Emotional regulation	0.884	0.892	0.919	0.694
Mental resilience	0.908	0.918	0.935	0.730
Training intensity	0.854	0.960	0.883	0.699
Wellbeing	0.871	0.878	0.898	0.666

### 5.4 Heterotrait-Monotrait (HTMT) ratio

Table 5 reports the Heterotrait-Monotrait (HTMT) ratio, a criterion used to assess discriminant validity by evaluating the degree to which constructs are distinct from each other. As recommended by Henseler et al. (2015), HTMT values below 0.85 indicate sufficient discriminant validity between constructs, while values above 0.85 suggest potential issues. In this table, all HTMT values are below the threshold of 0.85, except for a few close values, such as 0.809 between emotional regulation and mental resilience, and 0.768 between mental resilience and wellbeing. These values indicate that the constructs are mostly distinct, though some relationships, particularly between emotional regulation and mental resilience, are moderately strong. Nonetheless, the overall results support the discriminant validity of the model, indicating that the constructs are sufficiently different from each other to be considered unique.

**TABLE 5:** Heterotrait-monotrait (HTMT) ratio.

Construct	EF	PF	ER	MR	TI	WB
Emotion-focused						
Problem-focused	0.723	0.711	0.809	0.583	0.250	
Emotional regulation	0.752					
Mental resilience	0.558	0.632				
Training intensity	0.375	0.473	0.578			
Wellbeing	0.265	0.674	0.722	0.768		

EF, emotion-focused; PF, problem-focused; ER, emotional regulation; MR, mental resilience; TI, training intensity; WB, wellbeing.

In conclusion, the PLS-SEM results in these tables confirm the robustness of the measurement model, with no significant multicollinearity issues, strong reliability and validity of constructs, and acceptable levels of discriminant validity. These findings provide a solid foundation for further structural model analysis.

### 5.5 Structural model

Table 6 provides the path coefficients, *p*-values, *t*-values, and confidence intervals for the relationships between the variables in the model, including both direct effects, mediation effects, and moderated mediation effects. For the direct effects, both problem-focused coping and emotion-focused coping have a significant positive effect on wellbeing, with the path coefficient for problem-focused coping to wellbeing showing a value of 0.544 (*p* = 0.000, *t* = 10.411), and the path coefficient for emotion-focused coping to wellbeing having a value of 0.381 (*p* = 0.000, *t* = 6.424). These results indicate that both coping strategies significantly contribute to wellbeing, though problem-focused coping has a stronger direct impact than emotion-focused coping. Furthermore, the mediators, mental resilience and emotional regulation, both exhibit significant positive relationships with wellbeing, as shown by the path coefficients for mental resilience to wellbeing (0.449, *p* = 0.004) and emotional regulation to wellbeing (0.387, *p* = 0.000). These findings are in line with previous research, indicating that both resilience and emotional regulation play essential roles in maintaining athletes’ wellbeing (Fletcher and Sarkar, 2013; Gross, 1998).

**TABLE 6:** Path coefficients.

Path	*B*	*p*-value	*t*-value	Confidence intervals
Direct effects				
PF - > WB	0.544**	0.000	10.411	(0.462, 0.593)
EF - > WB	0.381**	0.000	6.424	(0.335, 0.468)
MR - > WB	0.449**	0.004	2.970	(0.356, 0.521)
ER - > WB	0.387**	0.000	6.423	(0.325, 0.466)
Mediators				
PF - > MR - > WB	0.176**	0.003	2.988	(0.115, 0.243)
EF - > MR - > WB	0.293**	0.000	5.689	(0.225, 0.364)
PF - > ER - > WB	0.314**	0.000	7.091	(0.249, 0.382)
EF - > ER - > WB	0.285**	0.006	2.881	(0.214, 0.356)
Moderator				
PF x TI - > MR - > WB	0.154**	0.000	5.442	(0.101 0.215)
EF x TI - > MR - > WB	0.212**	0.010	2.110	(0.161, 0.269)
PF x TI - > ER - > WB	0.198**	0.000	6.434	(0.135, 0.258)
EF x TI - > ER - > WB	0.176**	0.000	8.347	(0.110, 0.248)

EF, emotion-focused; PF, problem-focused; ER, emotional regulation; MR, mental resilience; TI, training intensity; WB, wellbeing. **Correlations are significant at 0.01 level (two-tailed).

For the mediated effects, the results reveal that both mental resilience and emotional regulation mediate the relationships between coping strategies and wellbeing. The indirect effect of problem-focused coping on wellbeing through mental resilience was significant [βββ = 0.176, *t* = 2.988, *p* = 0.003, 95% CI (0.115, 0.243)]. Likewise, the indirect effect through emotional regulation was significant [β = 0.314, *t* = 7.091, *p* = 0.000, 95% CI (0.249, 0.382)]. Similarly, emotion-focused coping exerted significant indirect effects on wellbeing through mental resilience [β = 0.293, *p* = 0.000, 95% CI (0.225, 0.364)] and emotional regulation [β = 0.285, *p* = 0.006, 95% CI (0.214, 0.356)]. As none of the confidence intervals included zero, the mediation effects were supported. These findings are consistent with prior studies emphasizing the mediating roles of resilience and emotional regulation in the stress-coping process (Tugade and Fredrickson, 2004).

**FIGURE 2:**
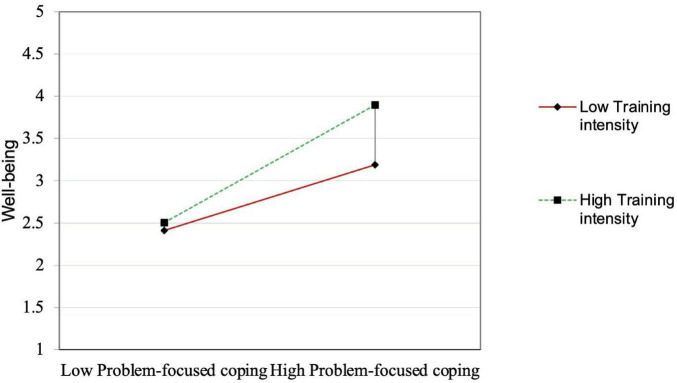
Moderated mediation effect of training intensity on the association between problem-focused coping and wellbeing mediating by mental resilience.

**FIGURE 3:**
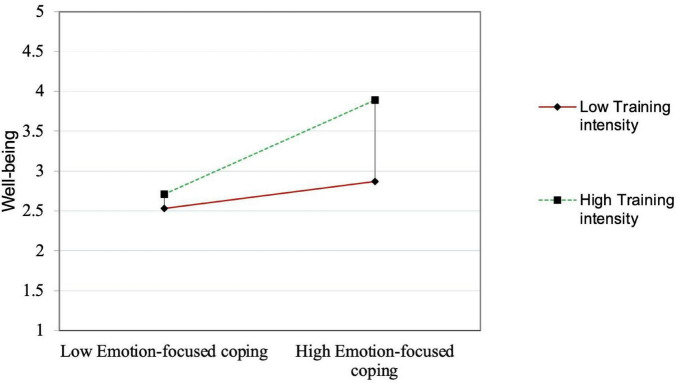
Moderated mediation effect of training intensity on the association between emotion-focused coping and wellbeing mediating by mental resilience.

**FIGURE 4:**
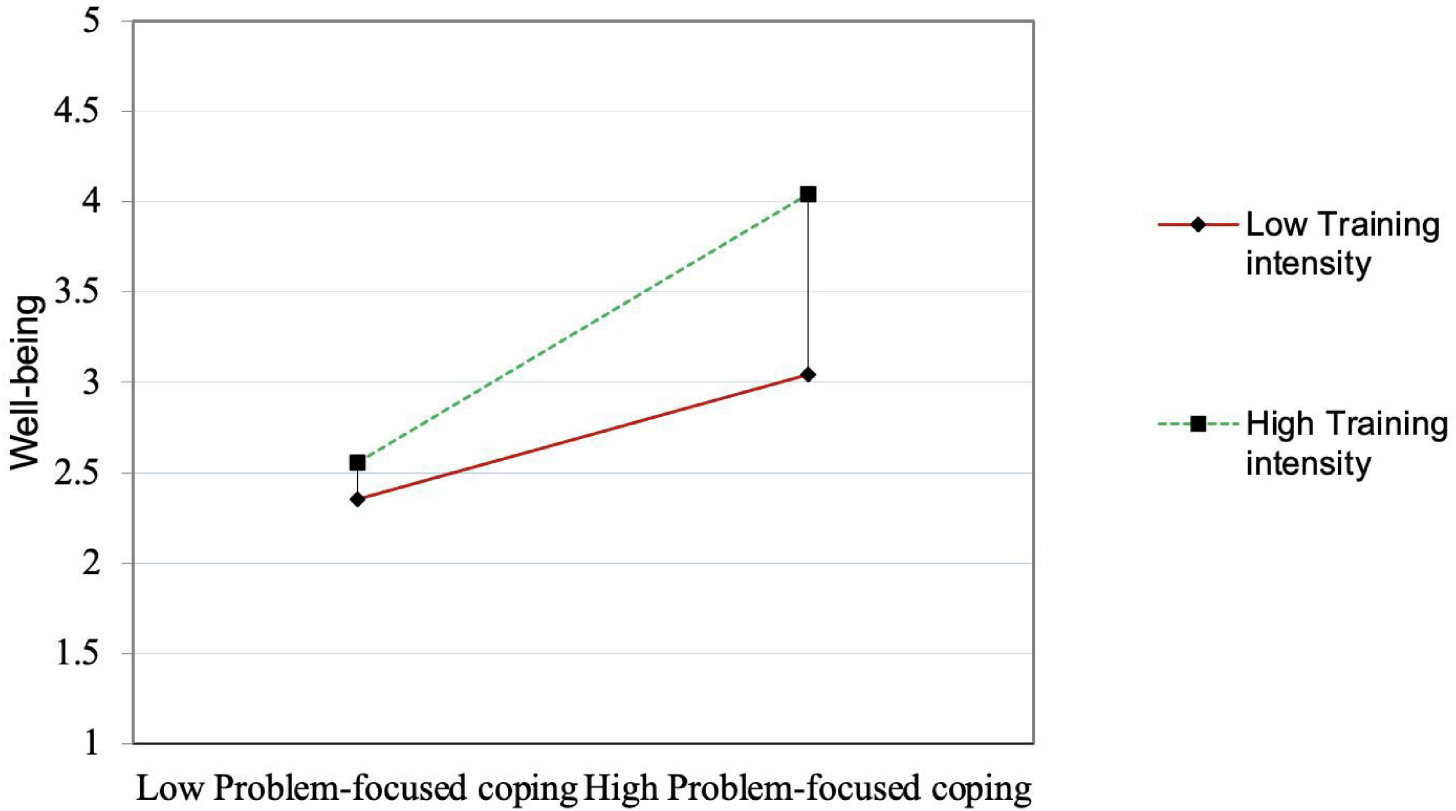
Moderated mediation effect of training intensity on the association between problem-focused coping and wellbeing mediating by emotional regulation.

**FIGURE 5:**
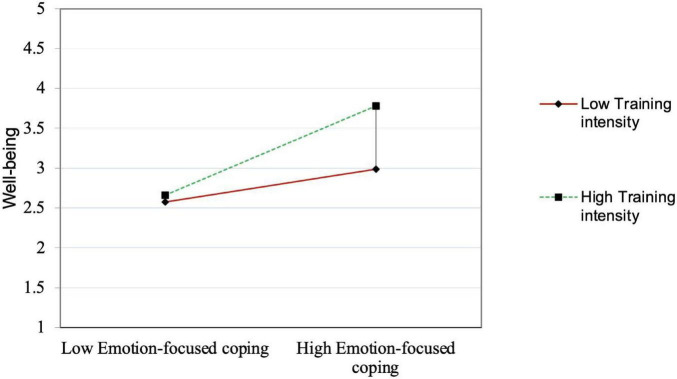
Moderated mediation effect of training intensity on the association between emotion-focused coping and wellbeing mediating by emotional regulation.

For the moderation effects, training intensity moderates the indirect relationships between coping strategies and wellbeing through both mental resilience and emotional regulation. The interaction effects of problem-focused coping multiplied by training intensity to mental resilience to wellbeing (0.154, *p* = 0.000) and problem-focused coping multiplied by training intensity to emotional regulation to wellbeing (0.198, *p* = 0.000) show that training intensity amplifies the positive effects of problem-focused coping on wellbeing through both mediators. Likewise, for emotion-focused coping, the interaction effect of emotion-focused coping multiplied by training intensity to mental resilience to wellbeing is 0.212 (*p* = 0.010) and the interaction effect of emotion-focused coping multiplied by training intensity to emotional regulation to wellbeing is 0.176 (*p* = 0.000), indicating that higher training intensity strengthens the relationship between coping strategies and wellbeing, mediated by both resilience and emotional regulation. These findings are consistent with research suggesting that stressors like training intensity can intensify the need for effective coping strategies and amplify their benefits in high-pressure environments (Peters et al., 2017). The simple slope analyses are illustrated in Figures 2–5.

In summary, the path coefficients demonstrate that both problem-focused and emotion-focused coping strategies positively influence wellbeing, with mental resilience and emotional regulation serving as important mediators. Furthermore, training intensity moderates these mediated relationships, strengthening the impact of coping strategies on wellbeing in high-intensity training conditions. These results highlight the dynamic interplay between psychological coping mechanisms and environmental stressors in the context of athletic resilience.

## 6 Discussion of the findings

The primary objective of this study was to explore how coping strategies (both problem-focused and emotion-focused) are associated with wellbeing in athletes, with a particular focus on the mediating roles of mental resilience and emotional regulation, and the moderating role of training intensity. The study aimed to provide a comprehensive understanding of how these psychological factors are related to one another in high-intensity training environments, contributing to athletes’ overall wellbeing. Through the integration of the Transactional Model of Stress and Coping (Lazarus and Folkman, 1984), the study sought to elucidate the pathways through which athletes may manage stress and maintain mental health in demanding settings.

The findings indicate that the study’s objectives were successfully achieved. Both problem-focused and emotion-focused coping strategies were found to be positively associated with athletes’ wellbeing, mediated by mental resilience and emotional regulation. Additionally, training intensity moderated these relationships, with stronger indirect effects observed under higher-intensity training conditions. These results provide important insights into the associations among coping mechanisms, mental resilience, emotional regulation, and athletes’ wellbeing, particularly in environments where physical and psychological demands are elevated.

The results align with previous research, particularly regarding the positive association between problem-focused coping and wellbeing. Studies such as Madigan et al. (2020) and Yamaguchi et al. (2022) similarly found that athletes who employ problem-focused strategies to address training and performance challenges tend to report better psychological outcomes, including higher levels of wellbeing and lower burnout rates. In addition, the mediation effect of mental resilience corroborates findings from Shang and Yang’s (2021) mediating role of mental toughness, who highlighted resilience as a key factor associated with athletes’ ability to thrive under stress. Similarly, the role of emotional regulation as a mediator confirms prior research that has linked emotional regulation with improved wellbeing outcomes in high-pressure environments (Diotaiuti et al., 2023).

However, the results regarding emotion-focused coping show some divergence from previous literature. While emotion-focused strategies have often been associated with short-term relief rather than long-term wellbeing (Rose et al., 2023), the study found that, when mediated by emotional regulation, these strategies were positively associated with athletes’ wellbeing. This suggests that emotion-focused coping, when accompanied by effective emotional regulation, may be associated with more positive long-term wellbeing than previously thought. The ability to manage emotions under pressure appears to be closely associated with maintaining psychological balance (Szczypińska et al., 2021), especially in high-intensity training environments.

The moderating effect of training intensity also provides novel insights. While previous research has explored the impact of training intensity on physical performance (Spiering et al., 2021), fewer studies have examined how it interacts with psychological coping mechanisms. The findings suggest that under higher-intensity training conditions, the positive associations of both problem-focused and emotion-focused coping with wellbeing become stronger. This supports the notion that in more demanding environments, athletes may rely more heavily on their mental resilience and emotional regulation skills in relation to their wellbeing. These results extend the understanding of how training context is associated with the effectiveness of coping strategies and offer new directions for further research in this area.

In summary, the study contributes to the existing body of knowledge by confirming the positive associations among coping strategies, mental resilience, emotional regulation, and athletes’ wellbeing, while also providing new insights into the moderating effect of training intensity. These findings challenge some previous assumptions about emotion-focused coping and highlight the dynamic nature of coping processes in high-stress environments. Nevertheless, because the study employed a cross-sectional design, the observed relationships should be interpreted as associations rather than causal effects. Accordingly, reciprocal or bidirectional relationships—for example, the possibility that athletes with higher wellbeing are more likely to adopt adaptive coping strategies—cannot be ruled out and should be examined in future longitudinal or experimental research.

### 6.1 Research implications

This study offers several significant theoretical contributions. First, by applying the Transactional Model of Stress and Coping (Lazarus and Folkman, 1984) to the athletic context, the research broadens the scope of this model beyond general stress management frameworks to include resilience training in athletes. The study’s findings reinforce the model’s relevance, demonstrating that problem-focused and emotion-focused coping strategies are positively associated with wellbeing, mediated by mental resilience and emotional regulation. This theoretical extension highlights the model’s utility in high-performance, physically demanding contexts, where stressors are not only psychological but also physical in nature.

Moreover, this study deepens our understanding of the mediating roles of mental resilience and emotional regulation. While these factors have been explored in previous research (Diotaiuti et al., 2023; Oguntuase and Sun, 2022; Shang and Yang, 2021), this study integrates them into a cohesive model, showing how they function as psychological processes associated with the relationship between coping strategies and wellbeing. By empirically testing this mediation, the study provides robust support for the notion that mental resilience and emotional regulation represent important psychological mechanisms underlying the association between coping strategies and wellbeing.

Another key theoretical contribution is the study’s exploration of training intensity as a moderator. Previous research has largely overlooked how training environments may shape the effectiveness of coping strategies (Spiering et al., 2021), and this study addresses this gap by demonstrating that training intensity significantly moderates the association between coping strategies and wellbeing. The finding that both coping strategies exhibit stronger positive associations with wellbeing under higher-intensity conditions adds a new dimension to existing stress-coping frameworks, offering valuable insights into how context-dependent factors are associated with stress management.

The practical implications of this study are particularly relevant for coaches, sports psychologists, and athletes involved in high-performance training programs. First, the findings suggest that fostering both problem-focused and emotion-focused coping strategies is crucial for enhancing athletes’ wellbeing. Coaches and trainers should focus on developing athletes’ ability to not only address performance-related challenges (problem-focused coping) but also to effectively manage their emotional responses (emotion-focused coping) in stressful situations. Specifically, structured coping skills workshops and resilience training modules should be integrated into regular training schedules, with greater emphasis during peak training cycles and the weeks preceding major competitions, when training intensity and psychological demands are highest. This could be achieved through workshops, psychological skills training, or resilience-building activities that emphasize coping mechanisms.

Moreover, the identification of mental resilience and emotional regulation as key mediators provides practical avenues for intervention. Programs that aim to strengthen athletes’ resilience, such as resilience training or mental toughness exercises, can significantly enhance their capacity to cope with stress. Similarly, emotional regulation techniques, such as mindfulness or cognitive reappraisal strategies, should be integrated into training routines to help athletes manage the emotional toll of high-intensity competition. For example, coaches may schedule brief resilience-building sessions, guided reflection exercises, and mindfulness-based emotional regulation practices immediately before or after high-intensity training sessions to reinforce adaptive coping behaviors. By equipping athletes with these skills, teams can create more psychologically resilient athletes who are better prepared to handle the pressures of their sport.

The moderating effect of training intensity also offers actionable insights for training program design. Under high-intensity conditions, athletes rely more heavily on coping mechanisms, which means that support structures should be put in place during the most demanding phases of training. Accordingly, coaches and sport psychologists should implement structured resilience training modules, routine psychological monitoring, and periodic assessments of athletes’ coping capacity during periods of elevated training intensity to identify athletes requiring additional psychological support. Coaches can adjust training intensity based on athletes’ psychological readiness and provide additional support during peak periods, such as pre-competition phases. These findings also suggest that individualized training plans that account for both physical and psychological factors could optimize performance and wellbeing outcomes.

### 6.2 Limitations and future research directions

Despite the valuable insights provided by this study, several limitations should be acknowledged. First, the study relied on self-reported measures, which can be subject to bias, such as social desirability or inaccurate self-assessment of emotional states and coping strategies. Future research could address this limitation by incorporating objective performance data or physiological measures, such as heart rate variability or cortisol levels, to complement self-reports and provide a more comprehensive view of athletes’ stress and coping processes. Second, because the study employed a cross-sectional research design, the findings should be interpreted as evidence of associations rather than causal relationships. Consequently, reciprocal or bidirectional relationships among coping strategies, mental resilience, emotional regulation, training intensity, and wellbeing cannot be ruled out. Future studies should consider employing longitudinal or experimental designs to better capture the dynamic nature of coping strategies and wellbeing over time and to establish the temporal ordering of these relationships.

Another limitation is the focus on athletes in a specific training context, which may restrict the generalizability of the findings to other populations or environments. Future research could extend this model to different settings, such as corporate workplaces or academic environments, where individuals face varying types of stressors. Moreover, exploring additional moderators, such as individual differences in personality traits (e.g., optimism or neuroticism) or team dynamics, could provide further insight into how coping strategies and wellbeing interact across different contexts. Research could also examine how cultural factors influence the effectiveness of coping strategies and emotional regulation in managing stress, offering a more nuanced understanding of these processes globally.

## 7 Conclusion

The study provides valuable insights into the relationships between coping strategies, mental resilience, emotional regulation, and wellbeing in athletes, while highlighting the moderating role of training intensity. The findings demonstrate that both problem-focused and emotion-focused coping strategies are positively associated with wellbeing, with mental resilience and emotional regulation acting as key mediators in this process. Additionally, the moderating effect of training intensity highlights the importance of context in determining the strength of these associations. By examining these relationships, the study contributes to both theoretical understanding and practical applications for enhancing athletes’ psychological wellbeing, particularly in high-pressure training environments. Nevertheless, given the cross-sectional nature of the study, these findings should be interpreted as associative rather than causal, and future longitudinal or experimental research is needed to establish causal relationships among the study variables.

## Data Availability

The original contributions presented in the study are included in the article/supplementary material, further inquiries can be directed to the corresponding author.
